# Temporal trends in the primary prevention of implantable cardioverter-defibrillator selection and long-term outcomes in patients with non-ischaemic dilated cardiomyopathy and ischaemic cardiomyopathy

**DOI:** 10.1093/europace/euag062

**Published:** 2026-04-07

**Authors:** Thuy Mi Nguyen, Daniela Melichova, Eivind Westrum Aabel, Ivar Mjåland Salte, Erik Gjertsen, Geir Heggelund, Øyunn Kleiven, Harald Brunvand, Thor Edvardsen, Kristina Haugaa

**Affiliations:** ProCardio Center for Innovation, Department of Cardiology, Oslo University Hospital, Rikshospitalet Sognsvannsveien 20, PO Box 4950 Nydalen, Oslo NO-0424, Norway; Institute of Clinical Medicine, University of Oslo, PO Box 1171 Blindern, Oslo 0318, Norway; Department of Cardiology, Hospital of Southern Norway, Kristiansand 4604, Norway; ProCardio Center for Innovation, Department of Cardiology, Oslo University Hospital, Rikshospitalet Sognsvannsveien 20, PO Box 4950 Nydalen, Oslo NO-0424, Norway; Institute of Clinical Medicine, University of Oslo, PO Box 1171 Blindern, Oslo 0318, Norway; Department of Cardiology, Hospital of Southern Norway, Kristiansand 4604, Norway; ProCardio Center for Innovation, Department of Cardiology, Oslo University Hospital, Rikshospitalet Sognsvannsveien 20, PO Box 4950 Nydalen, Oslo NO-0424, Norway; Institute of Clinical Medicine, University of Oslo, PO Box 1171 Blindern, Oslo 0318, Norway; Institute of Clinical Medicine, University of Oslo, PO Box 1171 Blindern, Oslo 0318, Norway; Department of Cardiology, Hospital of Southern Norway, Kristiansand 4604, Norway; Department of Medicine, Drammen Hospital, Vestre Viken Hospital, Vestre Viken HF, PO Box 800, Drammen 3004, Norway; Department of Cardiology, University Hospital of North Norway HF, Postboks 100, Tromsø 9038, Norway; Department of Cardiology, Hospital of Southern Norway, Kristiansand 4604, Norway; Department of Cardiology, Helse Stavanger HF, Postboks 8100, Stavanger 4068, Norway; ProCardio Center for Innovation, Department of Cardiology, Oslo University Hospital, Rikshospitalet Sognsvannsveien 20, PO Box 4950 Nydalen, Oslo NO-0424, Norway; Department of Cardiology, Hospital of Southern Norway, Kristiansand 4604, Norway; ProCardio Center for Innovation, Department of Cardiology, Oslo University Hospital, Rikshospitalet Sognsvannsveien 20, PO Box 4950 Nydalen, Oslo NO-0424, Norway; Institute of Clinical Medicine, University of Oslo, PO Box 1171 Blindern, Oslo 0318, Norway; ProCardio Center for Innovation, Department of Cardiology, Oslo University Hospital, Rikshospitalet Sognsvannsveien 20, PO Box 4950 Nydalen, Oslo NO-0424, Norway; Institute of Clinical Medicine, University of Oslo, PO Box 1171 Blindern, Oslo 0318, Norway

**Keywords:** Sudden cardiac death, Implantable cardioverter-defibrillator, Non-ischaemic dilated cardiomyopathy, Ischaemic cardiomyopathy, Left ventricular ejection fraction, Ventricular tachycardia

## Abstract

**Aims:**

The effect of primary prevention ICD (ppICD) in non-ischaemic dilated cardiomyopathy (DCM) remains debated. We investigated long-term outcomes and the incidence of ppICD therapy in patients with DCM and ischaemic cardiomyopathy (ICM), comparing implants before and after 2017.

**Methods and results:**

We prospectively included ppICD patients from the multicentre IMPROVE study (2014–2022) with DCM and ICM. All underwent ECG and echocardiography at baseline. The primary outcome was appropriate ICD therapy; the secondary outcome was all-cause mortality. Outcomes were compared pre- and post-2017 in DCM and ICM patients. Among 393 ppICD patients (median age 66, 15% female), 115 had DCM (66 post-2017, 49 pre-2017) and 278 had ICM (165 post-2017, 113 pre-2017). DCM post-2017 patients were younger (54 vs. 64 years, *P* < 0.01) and had a higher left ventricular ejection fraction (LVEF) (33% vs. 24%, *P* < 0.001) compared to DCM pre-2017 patients. Nevertheless, ICD therapy [4.0 vs. 3.1/100 person-years (PY), *P* = 0.57] and all-cause mortality rates (2.0 vs. 4.7/100 PY, *P* = 0.09) were similar. ICM post-2017 patients had a slightly higher LVEF (31% vs. 29%, *P* = 0.04), fewer ICD therapies (3.1 vs. 6.2/100 PY, *P* = 0.01), and similar mortality rates (6.3 vs. 8.3/100 PY, *P* = 0.18) compared to pre-2017.

**Conclusion:**

Patient selection for ppICD in DCM changed post-2017, with a shift towards younger patients and better LVEF compared to those implanted pre-2017; however, appropriate ICD therapy and all-cause mortality rates remained unchanged. In ICM, lower ICD therapy rates post-2017 may reflect improvement in revascularization and heart failure treatments.

**Trial Registration:**

ClinicalTrials.gov Identifier: NCT02286908

## Graphical Abstract

**Graphical Abstract:**
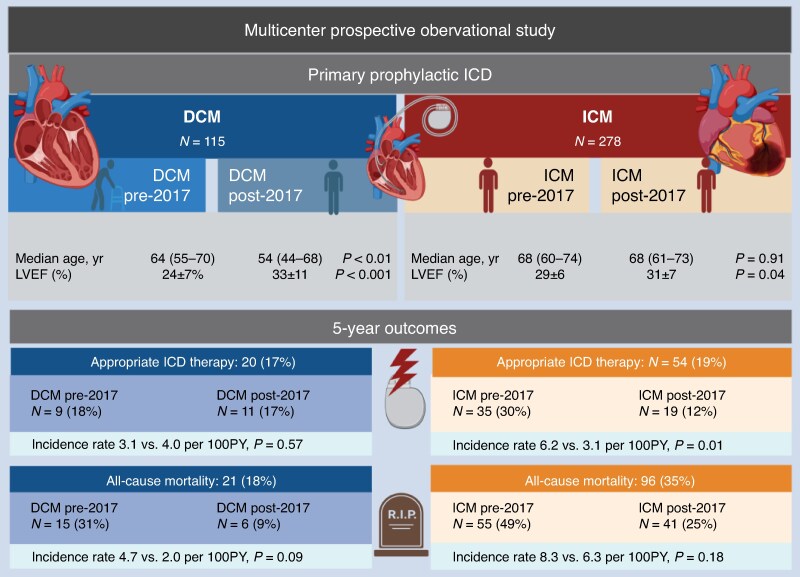

What’s new?

- Appropriate ICD therapy occurred in 19% of patients with ICM and DCM with ppICD during a median of 4.9 years of follow-up.
- DCM patients implanted with ppICD after 2017 were younger and had higher LVEF than those implanted before 2017, indicating a change in the selection of patients over time.
- DCM patients implanted with ppICD after 2017 had similar rates of ICD therapy and mortality compared to those implanted before 2017, indicating a high-risk population of ventricular arrhythmia.
- Fewer ICM patients after 2017 received appropriate ICD therapy, possibly reflecting advances in revascularization and heart failure management.
- Our study highlights the persistent risk of sudden arrhythmic death in patients with reduced LVEF, regardless of whether the underlying aetiology is DCM or ICM.

## Introduction

Identifying patients at high risk of sudden cardiac death (SCD) is a major clinical challenge and is paramount to ensure the success of primary prevention implantable cardioverter-defibrillator (ppICD) therapy. Patients with ischaemic cardiomyopathy (ICM) have generally had a poorer prognosis compared to those with non-ischaemic dilated cardiomyopathy (DCM).^1^ Both ICM and DCM patients had a class I recommendation for ppICD in the presence of symptomatic heart failure and LVEF ≤35% in the 2015 ESC Guidelines for the management of patients with ventricular arrhythmia and prevention of SCD.^2–5^. However, the findings from the DANISH trial (Defibrillator Implantation in Patients with Nonischemic Systolic Heart Failure), published in 2016, showed a limited benefit of ppICD in reducing all-cause mortality in patients with DCM.^6^ The results were explained by advancements in heart failure treatment that have improved survival rates in DCM, and the efficacy of ppICD therapy may have decreased accordingly. A 2017 survey of electrophysiologists conducted by the European Heart Rhythm Association highlighted a rapid and substantial influence of the DANISH trial on the clinical practices of ppICD implantation across most European countries.^7^ The 2022 ESC Guidelines for the management of patients with ventricular arrhythmias and the prevention of SCD adjusted the recommendation for ppICD implantation in DCM patients to class IIa and added several new recommendations for SCD risk stratification in subpopulations with DCM and increased SCD risk.^8^ However, few studies have examined how trends and changes in guidelines for implanting ppICD have affected the long-term outcomes of patients with DCM.

We aimed to explore long-term outcomes in patients with DCM and ICM who were implanted with ppICD from 2014 to 2022. Specifically, we aimed to investigate the rate of appropriate therapy from the ppICD and total mortality in DCM and ICM patients implanted before and after 2017 to assess potential changes in these populations. We hypothesized that the selection of DCM patients for ppICD has changed over the past ten years.

## Methods

### Study design, recruitment, and patient population

This study was part of the prospective, observational, multicentre, long-term follow-up IMPROVE study (ClinicalTrials.gov identifier: NCT02286908) conducted in Norway.^9,10^ The study aimed to include all consecutive patients who received ppICD implantation between 2014 and 2022 from five hospitals in Norway. Due to logistical constraints, a small number of eligible patients may have been unintentionally excluded. The inclusion criteria were age ≥ 18 years with indication for ppICD implantation. All patients were treated systematically according to the current guidelines at the time of inclusion.^5,8,11,12^ DCM was defined as dilated cardiomyopathy with reduced EF and no significant coronary artery stenosis on angiography, computed tomographic angiography, or nuclear myocardial perfusion imaging. ICM was defined as a prior history of MI, percutaneous coronary intervention (PCI), or coronary artery bypass grafting (CABG). Patient characteristics, biochemical work-up, electrocardiography (ECG), and echocardiography were prospectively obtained from hospital records. Cardiac magnetic resonance (CMR) and genetic testing were performed in selected cases of patients with DCM. Cardiac rhythm monitoring, including telemetry or Holter monitoring, was performed in indicated patients. Non-sustained ventricular tachycardia (nsVT) was defined as three or more consecutive ventricular beats, with a wide QRS complex and independent of atrial activity, occurring at a rate above 100 beats per minute, lasting less than 30 s, and terminating spontaneously without intervention.

Additional risk factors included nsVT on Holter monitoring, late gadolinium enhancement (LGE) on cardiac MRI, a history of syncope, and/or positive genetic testing. Exclusion criteria were a diagnosis of other specific cardiomyopathies or ion channelopathies.

The study was conducted by the Declaration of Helsinki and was approved by the Regional Committee for Medical Research Ethics (2013/573/REK Sør-Øst C). All study participants gave written informed consent.

### ICD implantation

ICD implantation was performed at the five recruiting centres, encompassing single- and dual-chamber devices, as well as cardiac resynchronization therapy with a defibrillator (CRT-D), as clinically indicated at the time of inclusion. Patients with existing conventional or CRT pacemakers who were eligible for an upgrade to a ppICD were included. Eligibility for ppICD implantation was determined through a comprehensive assessment of risk factors which included symptoms, medical treatment, ECG, blood tests, echocardiography, and arrhythmia risk assessment with Holter monitoring or telemetry. The study did not involve the registration of device-related complications post-implantation or during long-term follow-up. All patients were implanted with a transvenous ICD system. ICD programming was not part of the study protocol. Programming and follow-up were tailored to individual patients’ needs, at the discretion of treating cardiologists. However, in the subset of patients who had received ppICD, programming data were available before and after 2017.

### Long-term outcomes

The primary outcome was appropriate ICD therapy during follow-up, and the secondary outcome was all-cause mortality. Follow-up data for all outcomes were collected by December 2023 via medical record review and telephone calls at months 1, 3, and 6 after inclusion, and annually thereafter. The treating cardiologist examined all stored device therapy episodes. Appropriate ICD therapy was defined as sustained ventricular arrhythmia that was terminated with anti-tachycardia pacing (ATP) or shock therapy. Outcome data were collected from patients’ electronic medical records and were linked with the national death registry database.

### Echocardiography

Echocardiographic examinations were performed in all patients before the ICD implantation. The study examinations were performed using the Vivid 9.0 and Vivid 9.5 systems (GE Vingmed Ultrasound AS, Horten, Norway) and were analysed using commercially available software (EchoPAC v202–204, GE Healthcare, Horten, Norway). Three consecutive cardiac cycles in three apical planes and three short-axis planes were obtained using conventional 2D greyscale echocardiography with second harmonic imaging. Cine loops were digitally stored for subsequent offline analyses. Left ventricular ejection fraction (LVEF) was calculated from 4-chamber and 2-chamber images, using the modified Simpson´s rule.^13^ Strain measurements were performed using the semiautomatic speckle-tracking technique on standard two-dimensional echocardiographic images, analysed with EchoPAC software (GE Healthcare). LV global longitudinal strain (GLS) was assessed using speckle-tracking analysis of 2-dimensional (2D) greyscale image loops acquired at frame rates > 60 frames per second from three apical views. GLS was calculated as the average peak systolic strain in a 16-segment LV model.^14^

## Statistical analysis

Categorical variables were summarized as counts and percentages. Associations between groups were assessed using the chi-square test. Continuous data are presented as mean ± SD or as median [interquartile range (IQR)]. Comparisons of means were analysed using unpaired Student t-tests. For analysis of ICD events, the starting point (time zero) was defined as the date of device implantation. Absolute event incidence and incidence rates were calculated per 100 patients-years of follow-up and presented with 95% confidence interval (CI). Kaplan–Meier survival analysis was used to determine survival curves, and groups were compared using the log-rank test. Cox proportional hazards models were used to estimate both unadjusted and adjusted hazard ratios (HRs) (95% CI). *P*-values of <0.05 were considered statistically significant. All statistical analyses were performed using the Stata software package (Stata/SE version 17, StataCorp LLP, TX, USA).

## Results

### Study population

**Figure 1:**
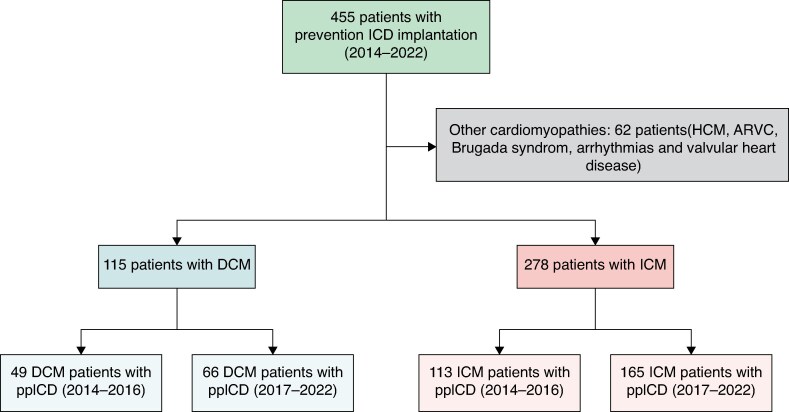
Flow chart, patients’ population. Details regarding device implantation in patients with non-ischaemic dilated cardiomyopathy (DCM) and ischaemic cardiomyopathy (ICM) before and after the DANISH trial. HCM = hypertrophic cardiomyopathy; ARVC = arrhythmogenic right ventricular cardiomyopathy; ppICD = primary prevention implantable cardioverter defibrillator. DCM pre-2017/ICM pre-2017 = patients included from 2014 to the end of 2016. DCM post-2017/ICM post-2017 = patients included from 2017 to 2022.

A total of 455 patients with indications for ppICD implantation were screened for inclusion in the study. Of these, 62 patients (14%) were excluded due to the presence of other specific cardiomyopathies or ion channelopathies (*Figure 1*). Consequently, 393 patients remained eligible for analysis, 115 (29%) with DCM and 278 (71%) with ICM. Among the DCM patients, 49 patients (43%) received ppICD between 2014 and 2016 (DCM pre-2017), and 66 patients (57%) received ppICD from 2017 to 2022 (DCM post-2017). Among the 278 patients with ICM, 113 (41%) received ppICD from 2014 to 2016 (ICM pre-2017), and 165 (59%) patients received ppICD from 2017 to 2022 (ICM post-2017).

#### Baseline characteristics in patients with DCM

The 115 DCM patients [77% men, median age 57 years (IQR 48–70)] had a LVEF of 30 ± 10%, and 18 (16%) received CRT-D. DCM patients post-2017 were younger (*P* < 0.01), had better LVEF (*P* < 0.001), better GLS (*P* = 0.02), shorter QTc (*P* < 0.001), and lower frequency of left bundle-branch blocks (LBBB) (*P* < 0.01 (*Table 1*) compared to DCM pre-2017. More DCM post-2017 ppICD patients (20%) were treated with sacubitril/valsartan (ARNI) compared with DCM pre-2017patients (6%) (*P* = 0.002).

**Table 1:** Baseline characteristics of patients with non-ischaemic dilated cardiomyopathy: a comparison between 2014–2016 and 2017–2022

	Prevention DCM ICD		
	DCM-ICD 2014–2016 (DCM pre-2017) *n* = 49	DCM-ICD 2017–2022 (DCM post-2017) *n* = 66	*P* value
Median age, yr. (IQR)	64(55–70)	54(44–68)	<0.01
Male, *n* (%)	39(78)	50(76)	0.63
**Median blood pressure (IQR)—mmHg**			
Systolic	122(110–130)	113(106–133)	0.41
Diastolic	75(65–80)	70(64–77)	0.39
Median body mass index, kg/m2(IQR**)** a	26.5(24.2–28.6)	26.0(23.6–32.4)	0.24
NT-proBNP, pg/mL (IQR)	1387(406–2723)	621(261–1785)	0.68
Creatinine, mg/dL (IQR)	90(74–111)	82(70–105)	0.84
Median estimated GFR, mL/min/1.73 m2 (IQR)	69(55–89)	81(62–98)	0.18
**12-lead electrocardiogram**			
Atrial fibrillation, *n* (%)	10(20)	16(24)	0.91
QRS, ms (IQR)	120(102–154)	111(101–125)	0.24
QTc, ms (IQR)	467(440–496)	441(419–465)	<0.001
Left bundle-branch block, *n* (%)	16(33)	5(8)	<0.01
Genetic testing, *n* = 37,			
Genotype-positive, *n* (%)	3(6)	12(18)	0.89
nsVT prior to ICD implantation, (%)	16(33)	33(50)	0.08
Cardiac MRI (*n* = 48)			
Late gadolinium enhancement on CMR	11(22)	18(27)	0.21
**NYHA class, *n* (%)**			
I	11(22)	26(39)	0.05
*I and additional risk factors* b	*10*(*20)*	*23*(*35)*	*0*.*11*
II	25(51)	28(42)	0.25
III-IV	11(22)	11(17)	0.37
**Coexisting conditions, *n* (%)**			
Hypertension	17(35)	21(32)	0.70
Diabetes	10(20)	9(14)	0.32
**Medication**			
ACE-I/ARB or ARNI	43(88)	50(76)	0.08
ARNI	3(6)	19(29)	0.002
Betablocker	45(92)	61(92)	0.89
Amiodarone	6(12)	6(9)	0.57
Mineralocorticoid receptor antagonist	23(47)	30(46)	0.82
Antiplatelet	3(6)	1(2)	0.18
Anticoagulant	21(43)	31(47)	0.72
Digoxin	6(12)	5(8)	0.91
Statin	29(59)	25(38)	0.02
SGLT2 inhibitor	0(0)	2(3)	0.22
CRTD, *n* (%)	14(30)	4(6)	<0.001
**Echocardiographic parameters (IQR)**			
LVEDV, ml	208(155–247)	197(134–246)	0.64
LVESV, ml	142(117–190)	129(83–174)	0.12
LVEF (mean, SD), %	24 ± 7	33 ± 11	<0.001
LVEF ≤ 35%, *n* (%)	47(96)	43(65)	<0.001
GLS, %	−7.8(−4.8 to −10.7)	−9.7(−6.7 to −12.2)	0.02

ACE-I, angiotensin-converting enzyme inhibitors; ARB, angiotensin-receptor blocker; ARNI, angiotensin receptor-neprilysin inhibitor; CMR, Cardiac Magnetic Resonance; CRTD, cardiac resynchronization therapy with defibrillator; DCM, non-ischaemic dilated cardiomyopathy; GFR, glomerular filtration rate; GLS, global longitudinal strain; ICD, implantable cardioverter-defibrillator; IQR, interquartile range; LVEF, Left ventricular ejection fraction; MD, mechanical dispersion; nsVT, non-sustained ventricular arrhythmias; NT-proBNP, *n*-terminal pro–brain natriuretic peptide; NYHA, New York Heart Association. SGLT2 inhibitor, Sodium–glucose cotransporter 2 inhibitor.

^a^The body mass index is the weight in kilograms divided by the square of the height in metres.

^b^NYHA class I with additional risk factors: nsVT on Holter monitoring, late gadolinium enhancement (LGE) on cardiac MRI, history of syncope, and/or positive genetic testing.

More DCM post-2017 patients with NYHA class I received a ppICD compared to pre-2017 (26 (39%) vs. 11 (22%), *P* = 0.05), and the frequency of additional risk factors was 35% vs. 20% (*P* = 0.11) in post- compared to pre-2017 patients (*Table 1*). Furthermore, there was a tendency for more nsVT to be detected on Holter or telemetry monitoring before ppICD implantation in DCM post-2017 patients compared to pre-2017 patients (50% vs. 33%) *P* = 0.08).

### Baseline characteristics in patients with ICM

The 278 ICM patients [89% men, median age 68 years (IQR 61–73)] had an LVEF of 30 ± 7%. Patients in the ICM post-2017 group had better LVEF (*P* = 0.04), similar GLS (*P* = 0.34), and lower NT-proBNP (*P* = 0.02) than the ICM pre-2017 group (*Table 2*). A significantly higher proportion of ICM post-2017 patients (23%) receiving ppICDs were treated with ARNI compared to ICM pre-2017 patients (2%) (*P* < 0.001).

**Table 2:** Baseline characteristics of patients with ischaemic cardiomyopathy: a comparison between 2014–2016 and 2017–2022

	Prevention ICM ICD		
	ICM-ICD 2014–2016 (ICM pre-2017)	ICM-ICD 2017–2022 (ICM post-2017)	*P* value
	*n* = 113	*n* = 165	
Median age, yr. (IQR)	68(60–74)	68(61–73)	0.91
Male, *n* (%)	101(89)	146(89)	0.82
Median blood pressure (IQR)—mmHg			
Systolic	120(110–130)	120(110–132)	0.96
Diastolic	74(67–80)	73(68–80)	0.55
Median body mass index, kg/m2(IQR**)** a	26.9(24.3–29.2)	27.8(24.9–31.3)	<0.01
NT-proBNP, pg/mL (IQR)	1615(457–4059)	889(415–1813)	0.02
Creatinine, mg/dL (IQR)	96(82–113)	95(81–115)	0.83
Median estimated GFR, mL/min/1.73 m2 (IQR)	66(51–80)	67(54–81)	0.52
**12-lead electrocardiogram**			
Atrial fibrillation, *n* (%)	20(18)	23(14)	0.90
QRS, ms (IQR)	110(98–126)	110(98–128)	0.56
QTc, ms (IQR)	438(418–469)	435(417–464)	0.07
Left bundle-branch block, *n* (%)	13(12)	15(9)	0.89
nsVT prior implantation	34(30)	60(36)	0.98
Cardiac MRI (*n* = 65)			
Late gadolinium enhancement on CMR	20(18)	40(24)	0.42
**NYHA class**, *n* (%)			
I	43(38)	49(30)	0.14
I and additional risk factors^b^	30(27)	41(25)	0.75
II	52(46)	83(50)	0.11
III-IV	16(14)	22(13)	0.88
**Coexisting conditions, *n* (%)**			
Hypertension	64(57)	90(55)	0.77
Diabetes	28(25)	50(30)	0.31
**Medication, *n* (%)**			
ACE-I/ARB or ARNI	90(80)	135(82)	0.65
ARNI	2(2)	38(23)	<0.001
Betablocker	109(96)	159(96)	0.97
Amiodarone	12(11)	18(11)	0.94
Mineralocorticoid receptor antagonist	58(51)	77(47)	0.45
Antiplatelet	46(41)	65(39)	0.83
Anticoagulant	50(44)	73(44)	0.99
Digoxin	6(5)	5(3)	0.48
Statin	108(96)	146(89)	0.05
SGLT2 inhibitor	0(0)	11(7)	0.005
CRTD, *n* (%) gjør likt som for meds	7(6)	4(2)	0.09
**Echocardiographic parameters (IQR)**			
LVEDV, ml	183(150–229)	167(134–221)	0.25
LVESV, ml	133(103–166)	116(93–152)	0.06
LVEF (mean, SD), %	29 ± 6	31 ± 7	0.04
LVEF ≤ 35%, *n* (%)	108(96)	139(84)	<0.01
GLS, %	−8.8(− 4.9 to −10.9)	−8.2(−4.0 to −10.3)	0.34

ACE-I, angiotensin-converting enzyme inhibitors; ARB, angiotensin-receptor blocker; ARNI, Angiotensin receptor–neprilysin inhibitor; CMR, Cardiac Magnetic Resonance. CRTD, cardiac resynchronization therapy with defibrillator; GFR, glomerular filtration rate; GLS, global longitudinal strain; ICD, implantable cardioverter-defibrillator; ICM, ischaemic cardiomyopathy; IQR, interquartile range; LVEF, Left ventricular ejection fraction; MD, mechanical dispersion; nsVT, non-sustained ventricular arrhythmias; NT-proBNP, *n*-terminal pro–brain natriuretic peptide; NYHA, New York Heart Association. SGLT2 inhibitor, Sodium–glucose cotransporter 2 inhibitor.

^a^The body mass index is the weight in kilograms divided by the square of the height in metres.

^b^NYHA class I with additional risk factors: nsVT on Holter monitoring, late gadolinium enhancement (LGE) on cardiac MRI, history of syncope, and/or positive genetic testing.

Among ICM patients with NYHA class I, the proportion receiving a ppICD did not differ between post- and pre-2017 patients (49 (30%) vs. 43 (38%), *P* = 0.14). The frequency of additional risk factors was also similar in post-2017 compared with pre-2017 patients (41 (25%) vs. 30 (27%), *P* = 0.75) (*Table 2*).

### Outcomes

#### Outcomes in the total population

Patients were followed for a median of 4.9 years [IQR 3.2–6.7]. The primary outcome of appropriate ICD therapy occurred in 74 patients (19%) [4.3 events per 100 person-years (PY)], of whom 59 patients (15%) received shock therapy. A total of 117 patients died during follow-up, resulting in an overall mortality rate of 30%. The cause of death was SCD in 58 patients (50%), non-sudden cardiovascular death in 42 patients (36%), and non-cardiac death in 17 patients (15%). No patients were lost to follow-up for the all-cause mortality analyses. Among patients who received ICD therapy and had available ICD programming data, our limited data showed no obvious changes in device programming before or after 2017 in either the DCM or ICM groups (see Supplementary material online, *Table S6*).

#### Outcomes in patients with DCM

In patients with DCM, appropriate ICD therapy occurred in 20 patients (17%) with a similar incidence rate in the DCM post-2017 and DCM pre-2017 (17% vs. 18%, 4.0 events/100 PY vs. 3.1 events/100 PY, *P* = 0.57). Total mortality occurred in 21 patients (18%), SCD in 9 patients (5%), other cardiac death in 10 patients (6%), and non-cardiovascular causes in 2 patients (2%). Cause-specific mortality was similar in the DCM post- and DCM pre-2017 patients (*Table 3*).

**Table 3:** Appropriate ICD therapy and all-cause mortality in patients with non-ischaemic dilated cardiomyopathy

	Total *n* = 115	DCM pre-2017 *n* = 49	DCM post-2017 *n* = 66	Hazard Ratio (95% CI)	*P* value
**Appropriate ICD therapy**					
Both ATP and shock, *n* (%)	20(17)	9(18)	11(17	1.17(0.74–1.85)	0.50
ATP from ICD, *n* (%)^a^	17(15)	6(12)	11(17)	1.51(0.88–2.58)	0.13
Sustained VT requiring shock from ICD, *n* (%)	17(15)	6(14)	11(14)	1.46(0.85–2.51)	0.16
Appropriate ICD therapy > 1, *n* (%)	9(8)	5(10)	4(6)	1.11(0.55–2.24)	0.77
**Death from any cause, *n* (%)**	21(18)	15(31)	6(9)	0.72(0.44–1.18)	0.19
**Cardiovascular death (%)**					
Sudden cardiac death	9(5)	7(14)	2(3)	0.62(0.27–1.45)	0.27
Other cardiovascular death	10(6)	6(12)	4(6)	0.95(0.49−0.1.85)	0.89
Non-cardiovascular death	2(2)	2(4)	0(0)	1.43(0.841–2.43)	0.19
Death and/or appropriate ICD therapy, *n* (%)	38(33)	21(43)	17(26)	1.33(0.89–1.92)	0.17

ICD, implantable cardioverter defibrillator; *n*, number; DCM, non-ischaemic dilated cardiomyopathy; VT/VF, ventricular tachycardia/fibrillation.

^a^Receiving either ATP/burst or ATP/burst, followed by shock.

DCM pre-2017 = patients included from 2014 to the end of 2016.

DCM post-2017 = patients included from 2017 to 2022.

#### Outcomes in patients with ICM

**Figure 2:**
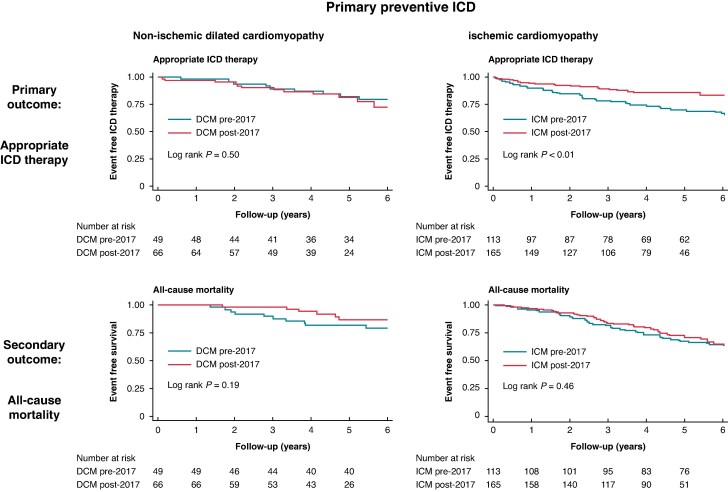
Survival analysis. Kaplan-Meier curves of appropriate ICD therapy and all-cause mortality in patients with non-ischaemic dilated cardiomyopathy (DCM) and ischaemic cardiomyopathy (ICM).

In patients with ICM, appropriate ICD therapy occurred in 54 patients (19%). The event rate was lower in ICM post-2017 compared to ICM pre-2017 patients (12% vs. 30%, 3.1 events/100 PY vs. 6.2 events/100 PY, *P* = 0.01) (*Figure 2*, *Tables 4* and *5*). Total mortality occurred in 96 ICM patients (35%), SCD in 49 patients (18%), other cardiac death in 32 patients (12%), and non-cardiovascular causes in 15 patients (5%), with no differences between ICM pre- and post-2017 (*Table 4*).

**Table 4:** Appropriate ICD therapy and all-cause mortality in patients with ischaemic cardiomyopathy

	Total *n* = 278	ICM pre-2017 *n* = 113	ICM post-2017 *n* = 165	Hazard Ratio (95% CI)	*P* value
**Appropriate ICD therapy**					
Both ATP and shock, *n* (%)	54(19)	35(30)	19(12)	0.66(0.49–0.87)	<0.01
ATP from ICD, *n* (%)^a^	29(10)	16(14)	13(8)	0.83(0.56–1.23)	0.36
Sustained VT requiring shock from ICD, *n* (%)	45(16)	30(27)	15(9)	0.61(0.44–0.83)	<0.01
Appropriate ICD therapy >1 time, *n* (%)	32(8)	22(20)	10(6)	0.58(0.40–0.86)	<0.01
**Death from any cause, *n* (%)**	96(35)	55(49)	41(25)	0.92(0.74–1.14)	0.46
**Cardiovascular death (%)**					
Sudden cardiac death	49(18)	33(29)	16(10)	0.78(0.56–1.07)	0.12
Other cardiovascular death	32(12)	14(12)	18(11)	1.22(0.84–1.76)	0.30
Non-cardiovascular death	15(5)	7(6)	7(4)	0.84(0.50–1.40)	0.50
Death and/or appropriate ICD therapy, *n* (%)	120(33)	67(59)	53(32)	1.08(0.88–1.32)	0.44

ICD, implantable cardioverter defibrillator; ICM, ischaemic cardiomyopathy; *n*, number; VT/VF, ventricular tachycardia/fibrillation.

^a^Receiving either ATP/burst or ATP/burst, followed by shock.

ICM pre-2017 = patients included from 2014 to the end of 2016.

ICM post-2017 = patients included from 2017 to 2022.

**Table 5:** Appropriate ICD therapy and all-cause mortality: incidence and incidence rates in patients with DCM and ICM patients pre- and post-2017

		Appropriate ICD therapy			All-cause mortality		
	Variable	Events	Incidence rate/100PY (95% CI)	*P* value	Events	Incidence rate/100PY (95% CI)	*P* value
		
	Total	74	4.3 (3.4–5.4)		117	6.0 (5.0–7.2)	
Sex	Female	7	2.4 (1.1–5.0)	0.08	13	4.3 (2.5–7.4)	0.16
	Male	67	4.7 (3.7–5.9)		104	6.4 (5.2–7.7)	
DEVICE	ICD	69	4.4 (3.5–5.6)	0.41	108	6.1 (5.1–7.4)	0.55
	CRTD	5	3.0 (1.2− 7.2)		9	4.9 (2.6–9.5)	
LVEF	LVEF > 35%	6	2.6 (1.1–5.7)	0.16	7	2.8 (1.4–5.9)	0.01
	LVEF ≤ 35%	68	4.5 (3.6–5.8)		110	6.5 (5.4–7.8)	
DCM	DCM pre-2017	9	3.1(1.6–5.9)	0.57	15	4.7 (2.8–7.6)	0.09
	DCM post-2017	11	4.0 (2.1–7.2)		6	2.0 (0.9–4.6)	
ICM	ICM pre-2017	35	6.2 (4.5–8.7)	0.01	55	8.3 (6.3–10.8)	0.18
	ICM post-2017	19	3.1 (2.0–4.9)		41	6.3 (4.6–8.5)	

CI, confidence interval; CRTD, cardiac resynchronization therapy with defibrillator; ICD, implantable cardioverter defibrillator; ICM, ischaemic cardiomyopathy; LVEF, left ventricular ejection fraction; DCM, non-ischaemic dilated cardiomyopathy; PY, patient year.

DCM pre-2017/ICM pre-2017 = patients included from 2014 to the end of 2016.

DCM post-2017/ICM post-2017 = patients included from 2017 to 2022.

## Discussion

This study demonstrated a high 5-year incidence of appropriate ICD therapy (19%) and a high total mortality of 30% in patients with DCM and ICM who received a ppICD between 2014 and 2022. Patients with DCM had a 5-year incidence of appropriate ICD therapy of 17% and a mortality of 18%, which remained unchanged post-2017, despite younger age and higher LVEF in patients implanted post-2017. These results indicate a shift in the selection of younger patients after 2017, particularly those at high risk of arrhythmia. In patients with ICM, the corresponding rates were19% and 35%, respectively, with a decline in ICD therapies but persistently high mortality post-2017. These findings underscore the continued risk of sudden arrhythmic death in patients with reduced LVEF, regardless of the underlying aetiology.

### Patients with DCM and ppICD

The 5-year incidences of 17% appropriate ICD therapy and 18% mortality were high in our DCM cohort. Our data are in line with the DANISH study reporting appropriate ICD therapy in 12% and all-cause mortality of 21%.^6^

DCM patients receiving ppICD post-2017 were on average 10 years younger and had better LVEF compared to those who were implanted with ppICD pre-2017. Importantly, the rate of appropriate ICD therapy remained high and unchanged in DCM post-2017, despite patients being younger and having better LVEF at implantation. Trends over the last decade have shifted from decisions based solely on LVEF ≤35% to incorporating other high-risk features, such as non-sustained ventricular arrhythmias on monitoring, genotype, syncope, and findings on CMR. One might speculate that these results indicate a better selection of DCM patients for ppICD post-2017, in whom a high arrhythmic risk may have outweighed younger age and better LVEF. In the DANISH trial, subgroup analysis indicated a benefit of ppICD in those aged ≤70 years with lower rates of SCD.^6^ This age-treatment interaction may indicate a more individualized decision-making process for younger patients.

Genetic testing in DCM has been evolving since the early 2000s, facilitating early detection of patients at risk by family screening.^15,16^ In Norway, genetic testing for DCM started in 2007 and was expanded and broadened from 2018,^17^ which may have influenced our results. Guidelines^8,18^ have increasingly stressed the ppICD indications in patients with high-risk genetic variants for DCM even when LVEF is >35% if specific additional risk factors are present. There was a tendency to more nsVT documented on arrhythmia monitoring pre-ICD implantation and a higher frequency of additional risk factors in DCM patients post-2017, indicating compliance with guidelines.

A moderate number of patients in our cohort had NYHA class I. Patients in NYHA class I are not explicitly included in the current guideline indications for ppICD.^19^ However, the guidelines emphasize other risk factors such as genotype, nsVT, CMR findings of fibrosis, syncope, or positive electrophysiological testing in risk stratification for ppICD, and these additional risk factors were frequently present in our cohort.

### Patients with ICM and ppICD

ICM patients post-2017 had slightly better LVEF and lower NT-pro-BNP levels than those pre-2017, indicating a slight trend to less severe disease in ICM ppICD patients over time. Importantly, the incidence of appropriate ICD therapies in ICM patients post-2017 decreased compared to those in ICM patients pre-2017. A low LVEF (≤35%) remains strongly associated with elevated risk of life-threatening arrhythmia and survival benefits of ICD therapy in ICM patients.^20^ The observed reduction in appropriate ICD therapy among ICM post-2017 patients may be related to overall improvements in the management of ischaemic heart disease and heart failure over time. Optimized revascularization strategies^10^ and advances in medical therapy reduce the risk of SCD in ICM patients.^21^ However, patients with persistently low LVEF remain at high risk, supporting the continued class Ia recommendation for ppICD implantation.^22^

The vast majority of ICM patients in our study received comprehensive heart failure treatment. Later years’ advancements in heart failure treatments have reduced rates of SCD and arrhythmic events^19,23,24^ and may have contributed to the lower rate of ppICD therapy post-2017.

### ICD programming and therapy outcomes

Contemporary ICD therapy, including modern programming strategies such as those demonstrated in the MADIT-RIT trial,^25^ which emphasizes higher rate cutoffs and prolonged detection intervals, has been shown to reduce inappropriate ICD therapies substantially and may influence clinical outcomes in patients evaluated for primary prevention ICD implantation. Our original study protocol did not include ICD programming, and available data on ICD programming were insufficient to test the impact of different ICD programming over time.

## Clinical implications

Our results underscore a continued high incidence of appropriate therapy from ppICDs in DCM and the importance of a comprehensive risk stratification for ppICD in patients with DCM. The results from the DANISH study should not be misinterpreted as indicating a lack of benefit of ppICD in patients with DCM. On the contrary, our results showed that younger DCM patients with better LVEF at the time of ppICD implantation had an unchanged rate of appropriate ICD therapy post-2017, indicating a continued need for ppICD in DCM. The shift in the DCM population selected for ppICD post- and pre-2017 was in line with the results from the EHRA survey.^7^ While the DANISH study suggested limited survival benefit from ppICD in DCM, selection of younger individuals with relatively preserved LVEF and high arrhythmic risk may enhance outcomes.

## Limitations

This study, based on prospective ppICD implantation across all types of heart failure, had a relatively small sample size, which may impact the power necessary to detect subtle differences in outcomes between the pre-2017 and post-2017 groups. As an observational study, there may be unmeasured confounding factors or selection biases that could influence the results, despite efforts to compare baseline characteristics between groups. Genetic testing evolved during the study period and differed between the five participating centres. Therefore, no conclusions can be drawn regarding the use of genetic testing.

ICD programming was not part of the study protocol. We acknowledge that MADIT-RIT–inspired programming and the introduction of home monitoring may have reduced the occurrence of ICD therapy and could have influenced our results. ICD programming data were available for only a subset of patients who received a ppICD, limiting our ability to assess potential differences in programming before and after 2017.

The lack of systematic collection of home-monitoring data represents a limitation, as it may have obscured potential differences in device management and therapy delivery over time, by preventing assessment of early clinician-led interventions such as optimization of ICD programming or adjustment of antiarrhythmic therapy following detection of increased arrhythmic burden.

Wearable cardioverter-defibrillators have not been used in Norway and were therefore not included in the present study. However, their increasing use over time may influence ICD implantation practices in other areas.^26^ None of our included patients received subcutaneous ICDs. Improved reliability and reduced lead-related complications associated with subcutaneous systems may increase their future use, especially among younger patients with DCM, and could influence implantation trends over time.^27,28^

## Conclusions

Appropriate ICD therapy (19%) and 5-year all-cause mortality rates (30%) were high in a ppICD cohort including DCM and ICM patients. Patient selection for ppICD in DCM changed over time, with post-2017 patients being younger and with higher LVEF. Despite this, DCM post-2017 had unchanged rates of appropriate ICD therapies and all-cause mortality compared to those implanted pre-2017. The unchanged high risk of arrhythmic events in DCM patients indicates that selection for ppICD may have moved from relying solely on LVEF to a more aetiology-specific strategy. Fewer ICM patients received appropriate ICD therapy post-2017, which may be related to overall improvements in the management of ischaemic heart disease and enhanced heart failure therapies over time.

## Supplementary Material

euag062_Supplementary_Data
