# Supplementary material for: Temporal trends in the primary prevention of implantable cardioverter-defibrillator selection and long-term outcomes in patients with non-ischaemic dilated cardiomyopathy and ischaemic cardiomyopathy
Source: Europace. 2026 Apr 7;28(4):euag062. doi: 10.1093/europace/euag062 (PMC13122353; doi:10.1093/europace/euag062)
Supplement: euag062_Supplementary_Data [file euag062_supplementary_data.docx]

## **Supplementary Table**

## **Table 6.** ICD programming and therapy in patients with DCM and ICM patients pre- and post-2017

| Appropriate ICD therapy *  (n =58) ** | VT/VF  cut-off rate | **Non-ischaemic dilated cardiomyopathy** | |  | **Ischaemic cardiomyopathy** | |  |
| --- | --- | --- | --- | --- | --- | --- | --- |
| ICD programming |  | DCM  pre-2017  (n=8) | DCM  post-2017  (n=9) | P value | ICM  pre-2017  (n=28) | ICM  post-2017  (n=13) | P value |
| **Ventricular tachycardia** |  |  |  |  |  |  |  |
| N (%) | <200 bpm | 5(63) | 2(22) | 0.15 | 18(64) | 8(62) | 1.0 |
| N (%) | ≥200 bpm | 3(37) | 7(78) | 0.15 | 10(36) | 5(39) | 1.0 |
| **Ventricular fibrillation** |  |  |  |  |  |  |  |
| N (%) | <230 bpm | 1(13) | 1(11) | 1.0 | 7(25) | 3(23) | 1.0 |
| N (%) | ≥230 bpm | 7(87) | 8(89) | 1.0 | 21(75) | 10(77) | 1.0 |

BPM = beats per minute; ICD = implantable cardioverter defibrillator; ICM = ischaemic cardiomyopathy; DCM = non-ischaemic dilated cardiomyopathy; VT = ventricular tachycardia; VF = ventricular fibrillation.

*Appropriate ICD therapy = ATP/burst or ATP/burst, followed by shock.

**Of the 74 patients who received appropriate ICD therapy, ICD programming data were available for 58.

Values represent the number of patients. P-values are from two-sided Fisher’s exact tests.

DCM pre-2017/ICM pre-2017 = patients included from 2014 to the end of 2016.

DCM post-2017/ICM post-2017 = patients included from 2017 to 2022.
